# Comparison of therapeutic effects of inhaled corticosteroids on three subtypes of cough variant asthma as classified by the impulse oscillometry system

**DOI:** 10.1186/s12931-019-1005-2

**Published:** 2019-02-26

**Authors:** Hiroyuki Sugawara, Atsushi Saito, Saori Yokoyama, Kazunori Tsunematsu, Hiroki Takahashi

**Affiliations:** 1 Sugawara Internal Medicine and Respiratory Clinic, Tomakomai, 053-0821 Japan; 2 Department of Respiratory Medicine and Allergology, Sapporo Medical University School of Medicine, Sapporo, Hokkaido 060-8543 Japan; 3 Department of Biochemistry, Sapporo Medical University School of Medicine, Sapporo, 060-8556 Japan

**Keywords:** Chronic cough, Cough variant asthma, Inhaled corticosteroids, Impulse oscillometry system, Pulmonary function test

## Abstract

**Background:**

Cough variant asthma (CVA) is one of the most common causes of chronic persistent cough, and early treatment with inhaled corticosteroids (ICSs) is recommended to attenuate the inflammation and remodeling. The impulse oscillometry system (IOS) is a novel device for respiratory functional assessment that has not yet been assessed in terms of CVA. Therefore, we investigated the relationship between CVA and IOS, and the difference in therapeutic effects of ICSs among the subtype classifications by IOS.

**Methods:**

The following ICSs were randomly prescribed in daily medical care: coarse-particle ICS (fluticasone propionate [FP]), fine-particle ICS (mometasone furoate [MF]), and moderate-particle ICS (budesonide [BUD]). Treatment effects were assessed by the Leicester cough questionnaire (LCQ) and were compared among three separated subtypes based on IOS measurements: central, peripheral, and resistless.

**Results:**

Regarding LCQ scores, in the central type, the LCQ of the MF group was significantly lower than FP and BUD. In the peripheral type, the LCQ of the FP group was significantly lower than MF and BUD. In the resistless type, the LCQ of the MF group was significantly lower than BUD. Also, IOS factors were improved by 4 weeks of therapy with ICS. Thus, there was strong relationship between subtypes and particle size in terms of effectiveness.

**Conclusions:**

There is a strong relationship between IOS subtype classification and ICS particle size in terms of therapeutic efficiency in CVA. It appears important to determine the ICS particle size, based on the IOS subtype classification, before treatment.

**Electronic supplementary material:**

The online version of this article (10.1186/s12931-019-1005-2) contains supplementary material, which is available to authorized users.

## Background

Cough is a very common complaint among patients with respiratory problems. Based on the duration of the cough, it is classified into persistent cough (more than 3 weeks, but less than 8 weeks) and chronic cough (8 weeks or longer). In Japan, it is reported that cough variant asthma (CVA) is the most common cause of chronic persistent cough, followed by sinobronchial syndrome, gastroesophageal reflux disease, atopic cough, and postinfectious cough [1–6]. To reach a final diagnosis of prolonged chronic cough, it is important to verify the initial therapies based on the patient’s history and laboratory data. However, it is not easy to diagnose the cause of cough and to select the best therapy in the clinic, given there is currently no gold standard for diagnosis. Spirometry is frequently used in respiratory medicine; however, it appears not to work as well for prolonged chronic cough, including CVA, as it does for asthma and chronic obstructive pulmonary disease. Thus, we focused on the impulse oscillometry system (IOS) as a result of the question that the treatment efficacy varies based on the measured value of IOS when examining patients with CVA in outpatient clinics. IOS, originally described by Dubois et al. [7], is a novel, noninvasive device for respiratory functional assessment using the forced oscillation technique. IOS uses sound waves to detect airway changes rapidly; these pressure signals separately quantify the degree of obstruction in the central and peripheral airways. In addition, IOS can determine respiratory resistant (R) and respiratory reactance (X) of both airways. Like spirometry, IOS values correlate with clinical symptoms and asthma control. Hence, we consider IOS a useful examination method to elucidate the condition of CVA. However, compared with spirometry, IOS is not a standard method as it is not used in many hospitals. Thus, this study aims to use IOS as a criterion for selecting the treatment method or evaluating the treatment effect for CVA.

Eosinophils are reportedly increased in the sputum, bronchoalveolar lavage (BAL) fluid, and bronchial mucosal tissue of patients with CVA, and the increase in eosinophils correlates with disease severity [7, 8]. Additionally, studies on biopsy specimens of central airway mucosa and BAL fluid recovered from peripheral airways and parenchyma have revealed that the degree of eosinophilia is similar between classic asthma and CVA [8]. Given CVA involves structural changes, such as subepithelial thickening, goblet cell hyperplasia, and vascular proliferation causing airway remodeling, CVA is considered to be a peripheral airway inflammatory disease similar to asthma [8]. Some cases of CVA will evolve into continuous asthma. Therefore, the early usage of inhaled corticosteroids (ICSs) is recommended as CVA treatment to attenuate the inflammation and remodeling. Recently, several particle sizes of ICS have been made commercially available. Some variation in therapeutic effect based on the particle size and the location of the inflammation has been suggested in asthma [9, 10]. On the other hand, which ICS particle sizes should be used for CVA treatment is a subject of debate. Therefore, we decided to examine the correlation between IOS and the therapeutic effect of ICS. IOS can be used to assess the parameters of large and small airways, and IOS values correlate better with clinical symptoms in asthma compared with spirometry [11] because IOS can detect subtle airway changes earlier than spirometry can [12]. Although the inflammation of the airways in CVA appears to extend from the central to the peripheral airways, [13] few studies have assessed the clinical application of IOS for the treatment of CVA. Respiratory system resistance at 5 Hz (R5) depicts general obstruction in both the central and peripheral airways, as lower vibration frequencies can be transmitted more distally in the lungs compared with higher frequencies. Conversely, the resistance of the respiratory system at 20 Hz (R20) is considered indicative of only the central airway, while the difference between R5 and R20 (R5–R20) is considered indicative of only the peripheral airway. Some studies have reported that the interpretation of the difference (R5–R20) between respiratory resistance (Rrs) values at 5 and 20 Hz remains debatable [14]. However, in this study, we analyzed by dividing participants into three groups with R20 and R5–R20 values, described above, to elucidate the discussion points. Thus, patients with CVA were classified into three subtypes: (a) high R20 type, which was defined as the central airway type; (b) high R5–R20 type, which was defined as the peripheral airway type; and (c) the type that neither R20 and R5–R20 is high, which was defined as the resistless airway type. Therefore, in this analysis, we investigated the clinical role of IOS in CVA using this subtype classification, including its utility as a tool for diagnosis and therapeutic evaluation, and the relationship between IOS values and ICS particle size.

## Methods

### Patients and treatments

This was a single-center, retrospective observational study at the Sugawara Internal Medicine and Respiratory Clinic in Japan. Based on medical record, all the consecutive and unselected patients with chronic persistent cough visited on the clinic between September 2015 and December 2016 were included. Chronic persistent cough was defined as a cough lasting more than 3 weeks without specific cause. In order to select the CVA patients, patients with postinfectious cough, GERD-associated cough, allergic rhinitis, atopic cough and bronchial asthma were excluded. Also, due to not continuing treatment in order to some reasons or diagnosed as other diseases after treatment, these patients dropped out. Several type of ICS were randomly prescribed by multiple physicians in daily medical care according to the Japanese Respiratory Society cough guidelines [3] as follows: fluticasone propionate group (FP: average particle size 4.1 μm [coarse particle]), mometasone furoate group (MF: average particle size 1.5 μm [fine particle]), or budesonide group (BUD: average particle size, 2.4 μm [moderate particle]) or others. Patients treated with ICS except for FP, MF, and BUD were excluded from this analysis because of small number. In addition to ICS treatment, all the patients were treated with salmeterol or formoterol (long-acting beta2-agonists [LABA]) and montelukast (leukotriene receptor antagonists [LTRA]). Therapeutic efficacy was assessed by the Leicester cough questionnaire (LCQ) at the baseline, 2 weeks, and 4 weeks after treatment. The study conformed to the principles outlined in the declaration of Helsinki and was performed with the approval of the institutional ethics committee of Sapporo Medical Association. Informed consent was obtained in the form of opt-out on the web-site.

### Measurements of IOS and pulmonary function

IOS measurements were obtained using a commercially available IO device (Master Screen IOS, Jaeger, Germany) according to the manufacturer’s recommendations [15]. The following values were obtained: resistance at 5 Hz (R5: indicates total airway resistance); resistance at 20 Hz (R20: approximates central airway resistance); the difference between R5 and R20 (R5-R20: considered to be an index for small airways); reactance at 5 Hz (X5: relates to compliance); resonant frequency (Fres); and integrated area of low-frequency X (AX) values [16–18]. Fres and AX are values that detect expiratory flow limitations.

After the IOS measurements, the MasterScreen IOS-Jaeger (Germany) device was used to perform spirometry. To avoid any negative effects of forced expiration on the airway, spirometry was never performed before the IOS measurements. The percent predicted forced vital capacity (%FVC), the percent predicted forced expiratory volume in 1 s (%FEV_1_), the FEV_1_/FVC ratio, the percent predicted the maximal mid-expiratory flow (%MMEF), and the percent predicted peak expiratory flow (%PEF) were obtained.

Equations reported by Vogel et al., given below, were used to calculate the predicted values of the IOS resistances: woman *R* = (3.14 + 0.0167 × age – 0.0403 × frequency) × 0.1 [kPa × l/s], men *R* = (2.4705 + 0.0109 × age – 0.02675 × frequency) × 0.1 [kPa × 1/s] [19]. Patients with diagnosed clinical CVA were divided into three groups as follows: the central type was defined as ≥100% of predicted R20 and < 100% predicted R5-R20. The peripheral type was defined as < 100% of predicted R20 and ≥ 100% of predicted R5-R20. The resistless type was defined as < 100% of predicted R20 and < 100% predicted R5-R20.

### LCQ score

LCQ is a questionnaire for the assessment of the Quality of Life in patients with chronic persistent cough, and is composed of three domains (Physical: 8 questions; Psychological: 7 questions; Social: 4 questions), and includes 19 items with a 7-point Likert response scale. LCQ takes 5–10 min to complete. Each item assesses symptoms, or the impact of symptoms, over the last 2 weeks on a 7-point Likert scale. Scores in three domains are calculated as the mean for each domain (range: 1–7). The total score (range: 3–21) is calculated by adding the domain scores together. High scores suggested a better quality of life. The questionnaire itself can be referred to in the previous paper [20]. This tool is brief, simple to administer, repeatable, and well validated. In this analysis, patients responded to the LCQ questionnaire while waiting for the consultation during every visit. Usage of the Japanese version of the LCQ has been approved by Surinder Birring, Akio Niimi, and Haruhiko Ogawa [21].

### Statistical analysis

The statistical analysis was performed on the basis of an intention-to-treat strategy. Numeric variables were expressed as means ± SEM. The differences between groups were tested by nonrepeated ANOVA with or without the Student–Newman–Keuls test. Comparison of differences before and after treatment was tested by a paired t-test or a Wilcoxon signed-rank test. Categorical variables were tested by the chi-squared test. A *P*-value less than .05 was considered statistically significant. Microsoft Excel 2007 (Microsoft Corporation, USA), the Excel Statistical Program File (ystat2008.xls, Igakutosho-shuppan Ltd., Tokyo, Japan), and GraphPad Prism v7 software (GraphPad, Inc., San Diego, CA, USA) were used for the data analysis and graph generation. Spearman’s correlation analysis and JMP13.0 (SAS Institute, Cary, NC, USA) were used to evaluate the coefficients of determination (ρ), residuals, and significance (p) to identify associations between pulmonary function tests and IOS indices.

## Results

### Patient selection

**Fig. 1:**
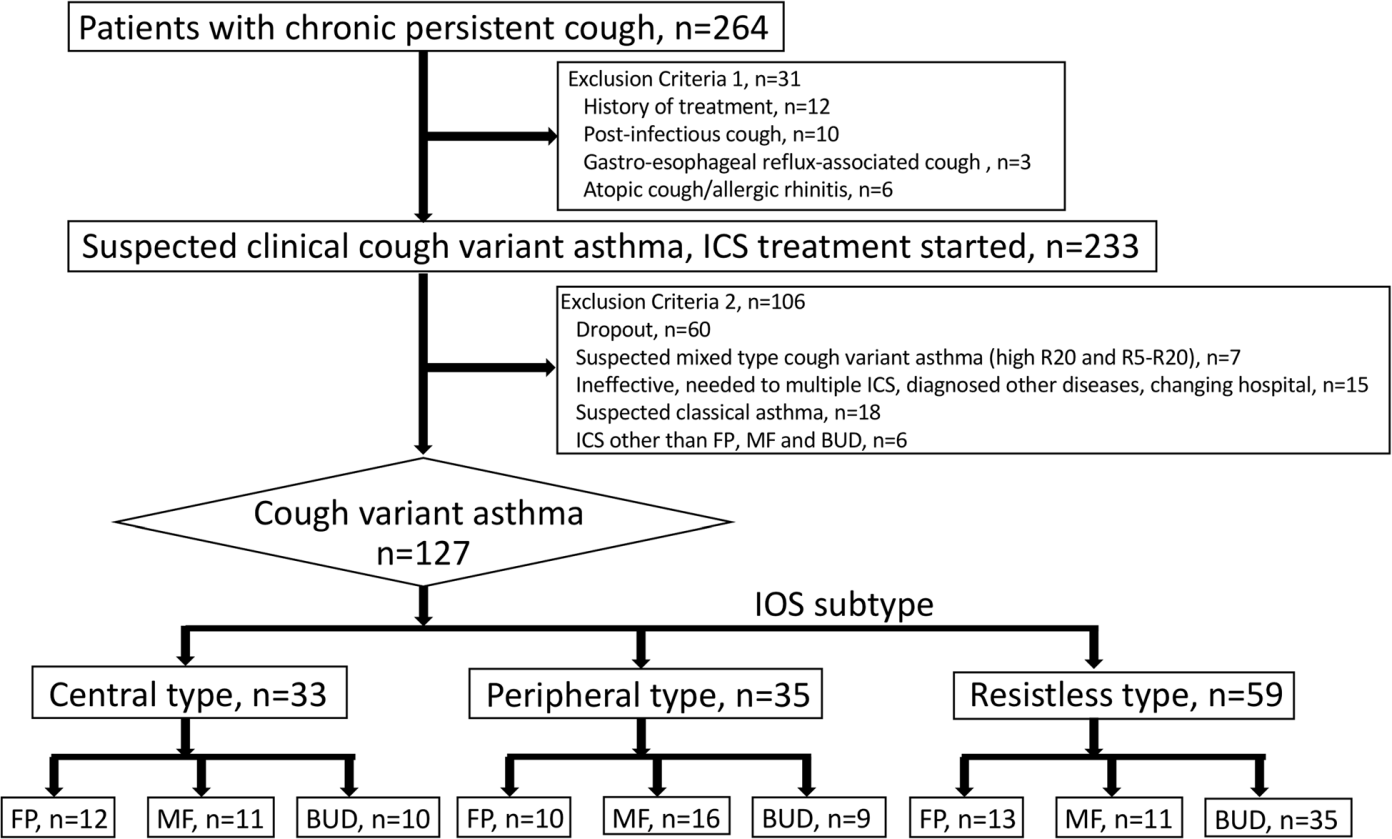
Flowchart. From 264 patients with persistent chronic cough, 127 patients with cough variant asthma participated in this analysis. A final total of 33 patients with central type, 35 patients with peripheral type, and 59 patients with resistless type were enrolled

Of 264 patients with persistent chronic cough screened, some patients were excluded with the exclusion criteria 1 and 2 as showed in Fig. 1. These patients were randomly treated with some kinds of ICS (FP, MF, BUD and others) with moderate dose. To compare the therapeutic effects of ICS on each IOS subtype as described below, the patients treated with small number of ICS except for FP, MF and BUD were also excluded. Finally, 127 patients who diagnosed as having CVA were enrolled in this analysis. Based on the results of the IOS, we divided the patients into three subtypes: central type (33 patients), peripheral type (35 patients), and resistless type (59 patients). The breakdown of ICS treatment was as follows; Central type (FP: 12, MF: 11, BUD: 10 patients, respectively), Peripheral type (FP: 10, MF: 16, BUD: 9 patients, respectively) and Resistless type (FP: 13, MF: 11, BUD: 35 patients, respectively) (Fig. 1).

### Baseline characteristics

Table 1 summarizes the characteristics of the patients. Compared with the other types, the patient’s age tended to be slightly lower in the central type. The peripheral types were more common among women and nonsmokers, and they had higher BMI values. On the other hand, men and smokers tended to be more common in the resistless type. Although no difference among the subtypes was observed in terms of %FVC, %FEV1, %MMEF and LCQ scores, the peripheral type had lower FEV1/FVC values compared with the central type, and %PEF was higher in the resistless type than in the other subtypes. Patient characteristics when divided into the three ICS groups are summarized in Additional file 1: Table S1. There were no statistically significant differences among the groups in spirometry values and LCQ scores.

**Table 1:** Baseline characteristics of the study patients

	All	Subtypes				Post-hoc test		
		Central	Peripheral	Resistless	^†^P	C-P	C-R	P-R
n	127	33	35	59				
Patient characteristic								
Age	45.7 (1.4)	39.4 (2.7)	50.4 (3.0)	46.7 (2.7)	*	*	*	NS
Male/Female (% Female)	46/81 (63%)	10/23 (70%)	3/32 (91%)	33/26 (44%)	**			
Atopy/Non-atopy (% Atopy)	56/50 (53%)	14/12 (53%)	14/19 (42%)	28/19 (59%)				
Duration of disease (Week)	5.3 (0.3)	5.6 (0.8)	5.0 (0.5)	5.2 (0.7)				
Smoker/Non-smoker (% Smoker)	52/75 (41%)	11/22 (33%)	10/25 (29%)	31/28 (53%)	*			
BMI	24.4 (0.4)	23.6 (0.7)	26.2 (0.8)	23.7 (0.7)	**	*	NS	*
Spirometry								
%FVC	103.4 (1.1)	100.6 (2.3)	104.0 (2.0)	105.0 (1.6)				
%FEV1	101.7 (1.1)	99.4 (2.2)	101.9 (2.3)	103.1 (1.4)				
FEV1/FVC	83.5 (0.5)	85.3 (1.2)	81.7 (0.8)	83.5 (0.8)	*	*	NS	NS
%MMEF	78.6 (1.9)	80.7 (4.0)	73.0 (3.4)	82.9 (2.5)				
%PEF	107.6 (1.4)	100.8 (2.3)	105.6 (3.0)	112.4 (1.8)	**	NS	**	*
Total LCQ score	11.8 (0.2)	12.1 (0.5)	11.6 (0.4)	11.8 (0.3)				

The data are shown as mean (SEM) or number (percentage). Differences among subtypes were tested by nonrepeated ANOVA (^†^P) with the Student–Newman–Keuls test for age, duration of disease, BMI, and spirometry (Post-hoc test). Differences in sex, atopy, and smoking were tested by the chi-squared test. Definition of abbreviations: *%FVC* Percent predicted FVC, *%FEV1* Percent predicted FEV1, *%MMEF* Percent predicted MMEF, *%PEF* Percent predicted PEF. *: *p* < .05, **: *p* < .01 and NS: not significant

### Therapeutic evaluation with LCQ

**Fig. 2:**
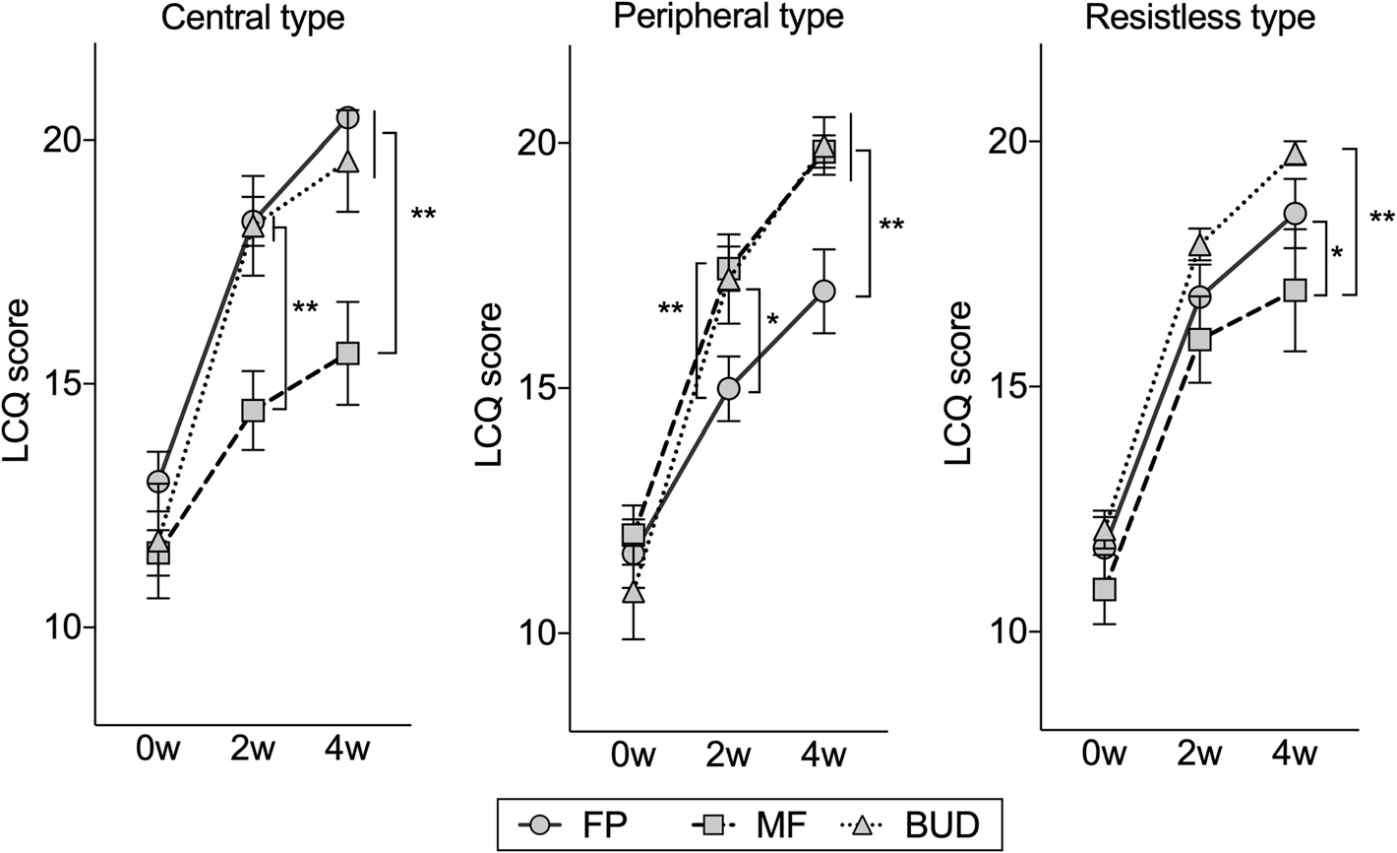
Comparison of LCQ scores in the ICS therapy groups in the subtypes at the baseline (0w) and after treatment (4w). FP: fluticasone propionate; MF: mometasone furoate; BUD: budesonide. Bars are expressed as the mean ± SEM. Differences among IOS groups were tested by nonrepeated ANOVA with Student–Newman–Keuls test. *: *p* < .05, **: *p* < .01

We evaluated the burden of cough in CVA, as well as patient-related outcomes with the LCQ, which is a simple and objective measure of cough. There were no statistically significant differences in terms of the LCQ between the three subtypes at baseline (Table 1). However, the LCQ scores after treatment in each subtype were quite different (Fig. 2). In the central type, the LCQ of the MF group was significantly lower than in the FP group (14.4 vs. 18.6, *p* < .01) and the BUD group (14.4 vs. 19.6, *p* < .01) at 2 weeks of treatment; and the FP group (15.6 vs. 20.5, *p* < .01) and the BUD group (15.6 vs. 18.3, *p* < .01) at 4 weeks of treatment. In the peripheral type, the LCQ of the FP group was significantly lower than in the MF group (15.0 vs. 17.8, *p* < .01) and the BUD group (15.0 vs. 17.4, *p* < .01) at 2 weeks of treatment; in the MF group (17.0 vs. 19.9, *p* < .01) and BUD group (17.0 vs. 19.8, *p* < .01) at 4 weeks. In the resistless type, the LCQ of the MF group was significantly lower than in the BUD group (16.1 vs. 17.9, *p* < .05) at 2 weeks of treatment; and in the BUD group (17.1 vs. 19.8, *p* < .01) at 4 weeks. These results indicate that improvements in patient symptoms are closely correlated with the particle size of the ICS. In other words, it appears possible to select an appropriate ICS by classifying patients by IOS prior to treatment.

### Therapeutic effects on pulmonary function and IOS factors

**Fig. 3:**
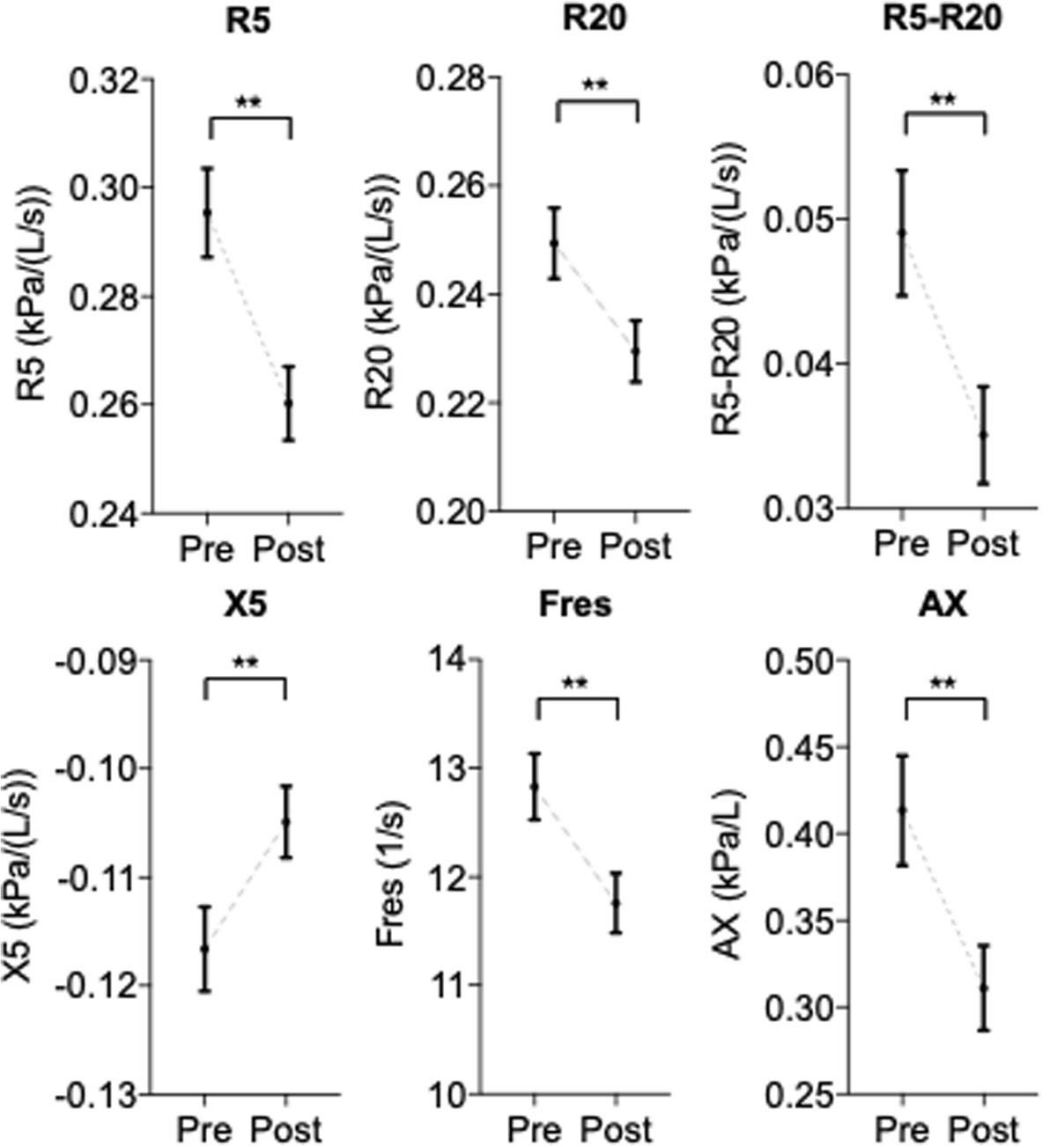
Comparison of impulse oscillometry index at baseline (Pre) and after treatment (Post). The data are expressed as the mean ± SEM. Differences between pre and post were tested by Wilcoxon signed-rank test. **: *p* < .01

**Fig. 4:**
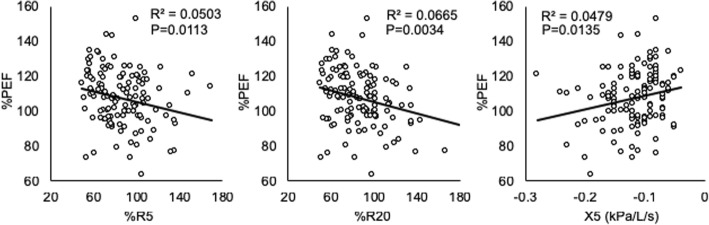
The relationships between %PEF in spirometry and %R5, %R20, and X5 in IOS. In each case, linear regression lines have been fitted. Spearman’s test. R^2^: Spearman rank-correlation coefficient

**Fig. 5:**
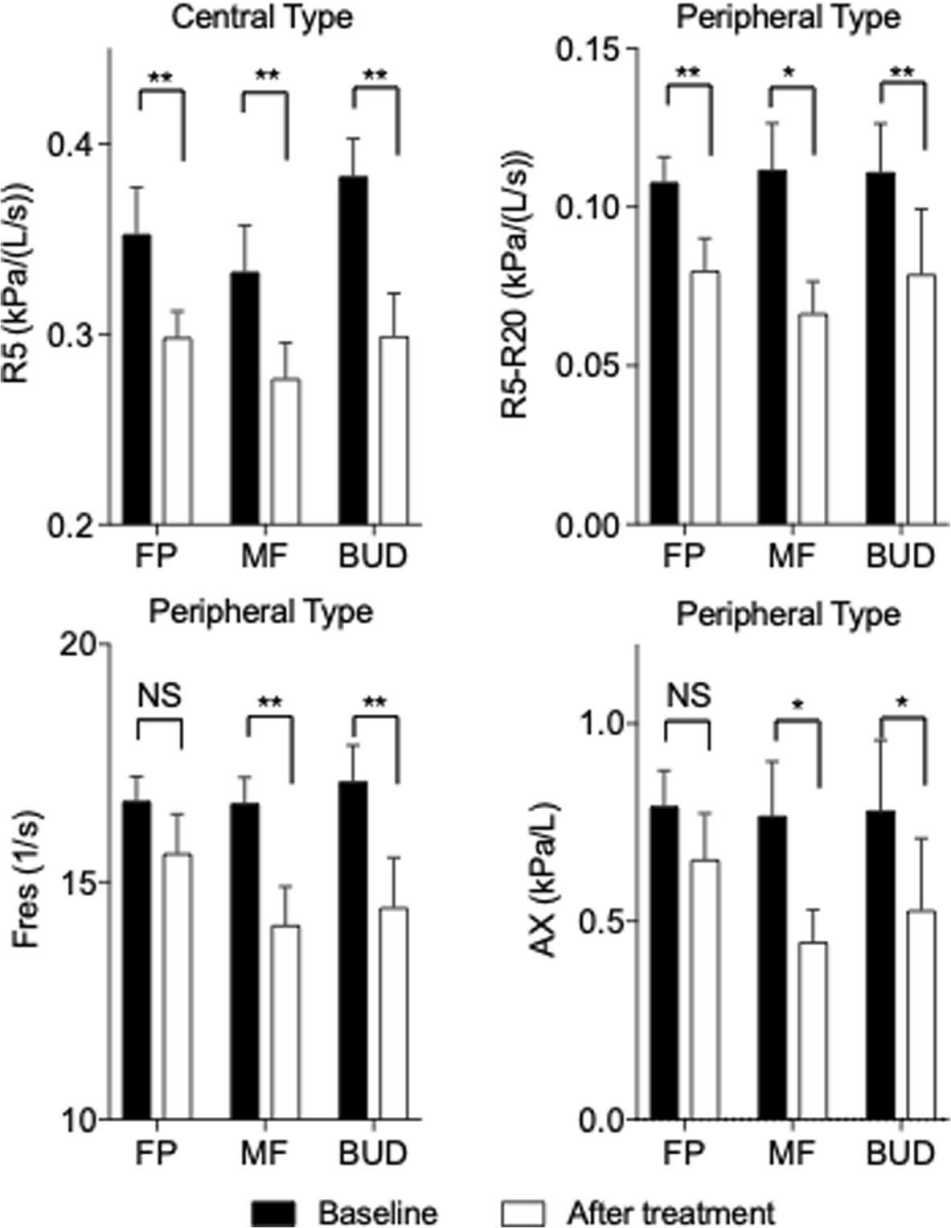
Comparison of IOS index in the ICS therapy groups in the central type and peripheral type at the baseline and after treatment. Bars are expressed as the mean ± SEM. Refer to Additional file 1: Table S2 for the original data. Differences between the baseline and after treatment were tested by a paired t-test. *: *p* < .05, **: *p* < .01, NS; not significant

Next, we examined whether IOS factors were related to the therapeutic effects. Regarding pulmonary function tests, there were no significant differences among the three subtypes at both baseline and post-treatment, except for %PEF (Table 2). Regarding the IOS factors, when we analyzed all the cases together, most IOS factors were improved at 4 weeks of therapy (Fig. 3). Additionally, there was a strong relationship between %PEF and %R5, %R20 and X5 (Fig. 4). Analyzing each of the three ICS groups individually, both R5 in the central type and R5-R20 in the peripheral type were significantly improved by treatment with any particle size of ICS. On the other hand, small-particle ICSs such as MF and BUD, but not FP, significantly decreased the index of Fres and AX, which appear to represent obstructive or restrictive impairments of the distal respiratory tract (Fig. 5). These results indicate that IOS indices might help us understand the effect of treatment with ICSs in CVA.

**Table 2:** Comparison of impulse oscillometry and spirometry over subtypes at the baseline (Pre) and after treatment (Post)

	Subtypes				Post-hoc test		
	Central	Peripheral	Resistless	^‡^P	C-P	C-R	P-R
Impulse oscillometry							
R5 (kPa/L/s)							
Pre	0.36 (0.01)	0.36 (0.01)	0.22 (0.01)	**	NS	**	*
Post	0.29 (0.01)	0.31 (0.01)	0.21 (0.01)				
^†^P	**	*	NS				
R20 (kPa/L/s)							
Pre	0.33 (0.01)	0.25 (0.01)	0.20 (0.01)	**	**	**	**
Post	0.28 (0.01)	0.24 (0.01)	0.19 (0.01)				
^†^P	**	NS	NS				
R5-R20 (kPa/L/s)							
Pre	0.03 (0.01)	0.11 (0.01)	0.03 (0.01)	**	**	NS	**
Post	0.02 (0.01)	0.07 (0.01)	0.02 (0.01)				
^†^P	NS	**	NS				
X5 (kPa/L/s)							
Pre	−0.12 (0.01)	−0.15 (0.01)	−0.10 (0.01)	**	**	NS	**
Post	−0.11 (0.01)	−0.13 (0.01)	−0.09 (0.01)				
^†^P	NS	NS	NS				
Fres (Hz)							
Pre	11.5 (0.5)	16.7 (0.3)	11.2 (0.3)	**	**	NS	**
Post	10.3 (0.3)	14.6 (0.5)	10.8 (0.3)				
^†^P	*	**	NS				
AX (kPa/L)							
Pre	0.32 (0.03)	0.78 (0.08)	0.25 (0.02)	**	**	NS	**
Post	0.24 (0.02)	0.53 (0.07)	0.21 (0.01)				
	**	**	**				
Spirometry							
%FVC							
Pre	100.6 (2.4)	104.0 (2.0)	105.0 (1.6)				
Post	102.8 (2.4)	107.6 (2.2)	107.1 (1.6)				
^†^P	NS	NS	NS				
%FEV1							
Pre	99.4 (2.2)	101.9 (2.3)	103.1 (1.4)				
Post	103.3 (2.3)	106.2 (2.5)	106.2 (1.5)				
^†^P	NS	NS	NS				
FEV1/FVC							
Pre	85.3 (1.2)	81.7 (0.8)	83.5 (0.8)	*	*	NS	NS
Post	86.9 (1.2)	82.1 (0.8)	84.3 (0.7)				
^†^P	NS	NS	NS				
%MMEF							
Pre	80.7 (4.0)	73.0 (3.4)	82.9 (2.5)				
Post	90.2 (4.4)	78.8 (3.7)	90.9 (2.9)				
^†^P	NS	NS	*				
%PEF							
Pre	100.8 (2.3)	105.6 (3.0)	112.4 (1.8)	**	NS	**	*
Post	113.2 (2.4)	119.8 (3.3)	123.4 (2.7)				
^†^P	**	**	**				

The data are shown as mean (SEM). Differences between the baseline and after treatment were analyzed by a paired t-test (^†^P). Differences among subtypes were tested by nonrepeated ANOVA (^‡^P) with the Student–Newman–Keuls test (Post-hoc test). C-P: between central type and peripheral type; C-R: between central type and resistless type; P-R: between peripheral type and resistless type. *: *p* < .05, **: *p* < .01 and NS: not significant

## Discussion

Multiple disorders cause chronic persistent cough. The cause of cough in Japan is CVA or asthma in 36% of cases, atopic cough in 29%, and sinobronchial syndrome in 17%, as reported by Fujimura [1]; Matsumoto reports 55% of cases are due to CVA/asthma, atopic cough in 15%, sinobronchial syndrome in 8%, and GERD in 7% [2]. From these reports, it appears that CVA is the most common cause of chronic persistent cough. Moreover, CVA is a precursor and a variant form of clinical asthma with typical symptoms of wheezing and dyspnea, unless adequate ICS treatment is provided [4, 22–24]. Additionally, previous studies have indicated the involvement of eosinophilic inflammation in CVA, as well as in classic asthma, as mentioned in the Introduction. Thus, it is especially important to use ICSs for the treatment of CVA. However, the most effective way to use ICSs in CVA treatment remains unclear. In this study, we have determined that subtype classification by IOS could be useful for the choice of ICS for treatment.

The values of the spirometry and health status (LCQ scores) at the baseline did not differ among the CVA subtypes and the ICS groups (Table 1). However, there were significant differences among subtypes when the efficacy of ICSs was evaluated by LCQ. Coarse-particle ICSs (FP) increased LCQ scores in the central type compared with other subtypes. Moreover, fine-particle ICSs (MF) increased the LCQ scores in the peripheral type (Fig. 2). These results indicate that coarse-particle ICSs are the most suitable for the central type of CVA and fine-particle ICSs are the most suitable for the peripheral type of CVA. On the other hand, moderate-particle ICSs (BUD) appear to be effective for both the central and peripheral types. Considering these results, we conclude that the differences in phenotypes in the IOS parameters are possibly related to the effectiveness of ICSs. In other words, the IOS examination should be performed for patients with CVA prior to ICS administration, and it is better to use ICS with a coarse-particle size for the central type and ICS with a fine-particle size for the peripheral type.

IOS measurements in persistent bronchial asthma correlate with clinical symptoms and asthma management [11, 25]. However, few studies on the correlation of IOS with spirometry have been reported [26–28]. Thus, it is thought that IOS is limited to being a diagnosis-supporting device. Small airways play an important role in persistent bronchial asthma. Lipworth identified persistent bronchial asthma with persistent small airway injury and poor control as a small airway asthma phenotype [29]. This is the first report to reveal the correlation between %PEF in spirometry and IOS parameters in CVA (Fig. 4). We believe that IOS is a useful device for the assessment of diseases located in peripheral airway, including CVA, because a slight change in such a lesion can be detected earlier with IOS than with spirometry [12, 30, 31].

Although there are only a few reports on the use of IOS for chronic persistent cough and CVA, it has been reported that the value of the baseline IOS did not differ significantly between patients with CVA and those with classic asthma, [32] and CVA indicated lower R5-R20, Fres, and AX, and higher X5 compared with classic asthma [33]. In addition, the level of R5 and R20 decreased in chronic persistent cough after treatment with ICSs and LABA, although there were no correlations between airway resistance and exhaled NO [34]. In addition to these reports, after treatment, R5 and R20 decreased in this analysis (Fig. 3). Thus, our observations appear to be compatible. This report is also the first on the subtype classification for CVA based on IOS parameters. We have observed that there was no difference in characteristics and spirometry among the subtypes (Table 1); however, the efficacy of ICS treatment correlated with the ICS particle size (Fig. 2). Thus, we conclude that IOS could be a useful device in the treatment of CVA.

Whether a correlation exists between the value of resistance in IOS and the pathological change remains unclear because of its less scientific basis. Regarding biopsy specimens of central airway mucosa and BAL fluid recovered from the peripheral airways, some studies have reported the presence of eosinophilic inflammation in the central and/or peripheral airway in patients with CVA [8]. In addition, structural changes secondary to airway inflammation have been reported in mucosal biopsies [35]. A computed tomography study revealed airway wall thickening in CVA [36]. Hence, we believe that the central airway-type or peripheral-type patients might have eosinophilic inflammation in the central or peripheral airways, respectively.

This study, as well as IOS, has some limitations. Spirometry is extensively adopted in studies for CVA or asthma. Being a novel methodology, IOS has not yet been adopted in many facilities, resulting in fewer evidence of the exact meaning, interpretation, and clinical application of IOS parameters compared with those of spirometry. As some data suggest that IOS should be used to evaluate the abnormal distal airway function, even in the standard spirometry setting, we believe that IOS could potentially detect the impairment of small or large airway function. As established normative values in different populations and comparability between different devices are lacking in IOS, developing robust reference values for IOS is imperative. In patients selection, we excluded chronic persistent cough that are not clearly CVA clinically and patients showing efficacy in LABA and ICS were diagnosed as CVA retrospectively. So, it is undeniable that the possibility of other cases of chronic persistent cough being mixed. In addition, CVA is common among children; however, children were not included in this study. Hence, pediatric cases should also be examined in the future trial. Although we mentioned the relationship between the particle size of ICS and IOS parameters, it is also true that the effectiveness of the three ICS devices cannot be simply determined by particle size. Also, it was a small, retrospective observational study. The patients were divided into three subtypes, in addition to the three types of ICS therapy; thus, the number of cases in each group was low. Furthermore, our follow-up period was short (mean observation period, 1.4 months). Long-term observations of CVA subtypes classified by IOS are needed to be able to predict how these phenomena will change over a longer follow-up.

## Conclusions

In conclusion, we have investigated the effect of treatment with ICSs and their relation to the parameters of IOS in patients with clinical CVA. Thus, phenotypic differences in IOS parameters could be associated with the efficacy of ICSs. Coarse-particle ICSs are effective for patients with central airway resistance, and fine-particle ICSs are effective for patients with peripheral airway resistance. Hence, we recommend the IOS examination for patients with CVA before the ICS administration. In addition, we found some variations in phenotype in the patients with CVA, such as bronchial asthma, and IOS could detect this slight change earlier than spirometry in peripheral lesions. Therefore, we have concluded that IOS can be useful for the selection of ICS for CVA treatment and the evaluation of CVA phenotype.

## Additional file

Additional file 1: **Table S1**. Characteristics and parameters over ICS group in subtypes. The data were showed as Mean (SEM) or number (Percentage). Definition of abbreviations is same as indicated in Table 1. **Table S2.** Comparison of impulse oscillometry and spirometry over ICS group in subtypes at baseline (Pre) and after treatment (Post). Definition of abbreviations is same as indicated in Table 1. The data were showed as Mean (SEM). Differences between baseline and after treatment were tested by un-paired t test (^†^P). Differences among IOS devices were tested by non-repeated ANOVA (^‡^P). *: *p* < .05, **: *p* < .01 and NS: no significant. (DOCX 149 kb)

## Abbreviations

AX: Area of low-frequency reactance
CVA: Cough variant asthma
FEV1: Forced Expiratory Volume in one second
Fres: Resonance frequency
FVC: Forced vital capacity
ICS: Inhaled corticosteroid
IOS: Impulse oscillometry system
LCQ: Leicester cough questionnaire
MMEF: Maximum mid-expiratory flow
PEF: Peak expiratory flow
R20: Resistance at 20 Hz
R5: Resistance at 5 Hz
R5-R20: Resistance at 5–20 Hz
X5: Reactance at 5 Hz
