# Supplementary material for: Comparison of therapeutic effects of inhaled corticosteroids on three subtypes of cough variant asthma as classified by the impulse oscillometry system
Source: Respir Res. 2019 Feb 26;20:41. doi: 10.1186/s12931-019-1005-2 (PMC6390374; doi:10.1186/s12931-019-1005-2)
Supplement: Supplementary file 1 — Table S1. Characteristics and parameters over ICS group in subtypes. The data were showed as Mean (SEM) or number (Percentage). Definition of abbreviations is same as indicated in Table 1. Table S2. Comparison of impulse oscillometry and spirometry over ICS group in subtypes at baseline (Pre) and after treatment (Post). Definition of abbreviations is same as indicated in Table 1. The data were showed as Mean (SEM). Differences between baseline and after treatment were tested by un-paired t test (†P). Differences among IOS devices were tested by non-repeated ANOVA (‡P). *: p < .05, **: p < .01 and NS: no significant. (DOCX 149 kb) [file 12931_2019_1005_MOESM1_ESM.docx]

**ADDITIONAL FILE**

**Comparison of therapeutic effects of inhaled corticosteroids on three subtypes of cough variant asthma as classified by the impulse oscillometry system**

Hiroyuki Sugawara MD, PhD, Atsushi Saito MD, PhD, Saori Yokoyama MD, Kazunori Tsunematsu MD, PhD, Hiroki Takahashi MD, PhD

**Table S1.**

**Table S2.**

**RESULTS**

**Table S1**. Characteristics and parameters over ICS group in subtypes. The data were showed as Mean (SEM) or number (Percentage). Definition of abbreviations is same as indicated in Table 1.

|  |  |  | Subtypes | | | | | | | | | | |
| --- | --- | --- | --- | --- | --- | --- | --- | --- | --- | --- | --- | --- | --- |
|  |  | Central | | | |  | Peripheral | | |  | Resistless | | |
|  |  |  | FP | MF | Bud |  | FP | MF | Bud |  | FP | MF | Bud |
| n | |  | 12 | 11 | 10 |  | 10 | 16 | 9 |  | 13 | 11 | 35 |
| Patient characteristic | |  |  |  |  |  |  |  |  |  |  |  |  |
|  | Age |  | 39.9 (1.5) | 37.1 (1.4) | 41.2 (1.2) |  | 57.8 (5.0) | 51.9 (4.1) | 39.4 (5.5) |  | 44.8 (4.2) | 39.7 (3.7) | 49.6 (2.6) |
|  | Male/Female (%Female) |  | 5/7 (58%) | 4/7 (63%) | 1/9 (90%) |  | 0/10 (100%) | 2/14 (88%) | 1/8 (89%) |  | 6/7 (54%) | 6/5 (46%) | 21/14 (40%) |
|  | Atopy/Non-atopy (%Atopy) |  | 7/5 (58%) | 2/6 (25%) | 5/1 (83%) |  | 2/ 8 (20%) | 7/7 (50%) | 5/4 (56%) |  | 8/3 (73%) | 6/3 (67%) | 14/13 (52%) |
|  | Duration of disease (weeks) |  | 5.6 (0.9) | 6.3 (2.2) | 4.8 (0.5) |  | 4.1 (0.3) | 5.6 (0.7) | 5.1 (1.0) |  | 4.5 (0.6) | 4.8 (0.9) | 5.6 (0.8) |
|  | Smoker/Non-smoker (%smoker) |  | 5/7 (42%) | 2/9 (18%) | 4/6 (40%) |  | 3/7 (30%) | 3/13 (19%) | 4/5 (44%) |  | 6/7 (46%) | 7/4 (64%) | 18/17 (51%) |
|  | BMI |  | 23.9 (1.0) | 22.8 (1.4) | 23.9 (1.5) |  | 24.7 (0.9) | 25.0 (1.6) | 27.8 (1.6) |  | 22.9 (0.9) | 23.3 (1.4) | 24.2 (0.6) |
| Spirometry | |  |  |  |  |  |  |  |  |  |  |  |  |
|  | %FVC |  | 95.1 (2.7) | 101.7 (3.8) | 106.1 (5.0) |  | 103.9 (3.7) | 106.1 (3.2) | 100.4 (3.4) |  | 105.2 (4.3) | 99.3 (3.0) | 106.2 (2.1) |
|  | %FEV1 |  | 95.4 (3.1) | 102.0 (4.4) | 101.3 (3.8) |  | 103.3 (5.1) | 103.0 (3.2) | 98.5 (4.3) |  | 101.7 (3.6) | 100.8 (3.8) | 104.1 (1.7) |
|  | FEV1/FVC |  | 86.7 (2.0) | 86.7 (2.2) | 82.1 (2.1) |  | 80.9 (1.2) | 80.7 (1.2) | 84.4 (1.6) |  | 82.4 (1.7) | 86.8 (1.9) | 83.0 (1.0) |
|  | %MMEF |  | 83.8 (5.3) | 85.4 (7.9) | 71.7 (7.2) |  | 75.7 (7.6) | 69.1 (4.4) | 76.8 (7.2) |  | 72.6 (3.0) | 87.7 (7.7) | 84.9 (3.3) |
|  | %PEF |  | 101.4 (3.7) | 98.2 (3.7) | 102.8 (5.3) |  | 103.9 (3.9) | 108.8 (5.3) | 101.7 (5.6) |  | 115.9 (3.2) | 109.1 (2.8) | 112.6 (2.8) |

**Table S2.** Comparison of impulse oscillometry and spirometry over ICS group in subtypes at baseline (Pre) and after treatment (Post). Definition of abbreviations is same as indicated in Table 1. The data were showed as Mean (SEM). Differences between baseline and after treatment were tested by un-paired t test (^†^P). Differences among IOS devices were tested by non-repeated ANOVA (^‡^P). *: p<.05, **: p<.01 and NS: no significant.
